# Supplementary material for: An electrochemiluminescence device powered by streaming potential for the detection of amines in flowing solution
Source: Nat Commun. 2025 Sep 8;16:8217. doi: 10.1038/s41467-025-63548-2 (PMC12417550; doi:10.1038/s41467-025-63548-2)
Supplement: Supplementary file 1 — Supplementary Information [file 41467_2025_63548_MOESM1_ESM.pdf]

Supplementary Information

An Electrochemiluminescence Device Powered by Streaming Potential for the Detection of Amines in Flowing Solution

Rintaro Suzuki,[1,#] Suguru Iwai,[1,#] Ryota Kirino,[1,#] Kosuke Sato,[1] Mariko Konishi,[1] George Hasegawa,[2] Norio Ishizuka,[3] Kimihiro Matsukawa,[4] Kazuo Tanaka,[5] Elena Villani[1,] and Shinsuke Inagi[1,]**

1. Department of Chemical Science and Engineering, School of Materials and Chemical Technology, Institute of Science Tokyo, 4259 Nagatsuta-cho, Midori-ku, Yokohama 226-8501, Japan
2. Institute of Materials and Systems for Sustainability, Nagoya University, Furo-cho, Chikusa-ku, Nagoya 464-8601, Japan
3. Emaus Kyoto, Inc., 26 Nishida-cho, Saiin, Ukyo-ku, Kyoto 615-0055, Japan
4. Materials Innovation Lab, Kyoto Institute of Technology, Goshokaido-cho, Matsugasaki, Sakyo-ku, Kyoto 606-8585, Japan
5. Department of Polymer Chemistry, Graduate School of Engineering, Kyoto University, Katsura, Nishikyo-ku, Kyoto 615-8510, Japan

# These authors contributed equally

* Corresponding authors: villani.e@mct.isct.ac.jp (E. V.)
inagi@cap.mac.titech.ac.jp (S. Inagi)

Table of Contents

1. Apparatus *S2*

2. Comparison of the two phenolic resin monoliths performances *S3*

3. Cell materials for streaming potential measurement *S4*

4. Formation of BTD-TPA coating onto the electrode *S6*

5. ECL generation and detection in the streaming potential system *S8*

6. Cyclic voltammetry and ECL measurements with a two-electrode system *S10*

7. Optimization of the amount of BTD-TPA coating onto the anode *S11*

8. Optimization of solvent composition *S13*

9. Electrolyte-free condition *S15*

10. Optimization of TPrA concentration *S17*

11. Impact of the electrode material on the ECL performances of the device *S19*

12. ECL imaging with a digital camera *S21*

13. ECL spectra measurement *S24*

14. Pulsed flow for the ECL system *S25*

15. Limit of detection (LOD) for TPrA in the device *S28*

16. Detection of other amines *S30*

17. Detection of aromatic amines *S32*

18. Aqueous systems (distilled water) *S33*

19. Aqueous systems (tap water) *S35*

20. Evaluation of the anti-interference capabilities of the device *S36*

21. Supplementary references *S39*

1. Apparatus

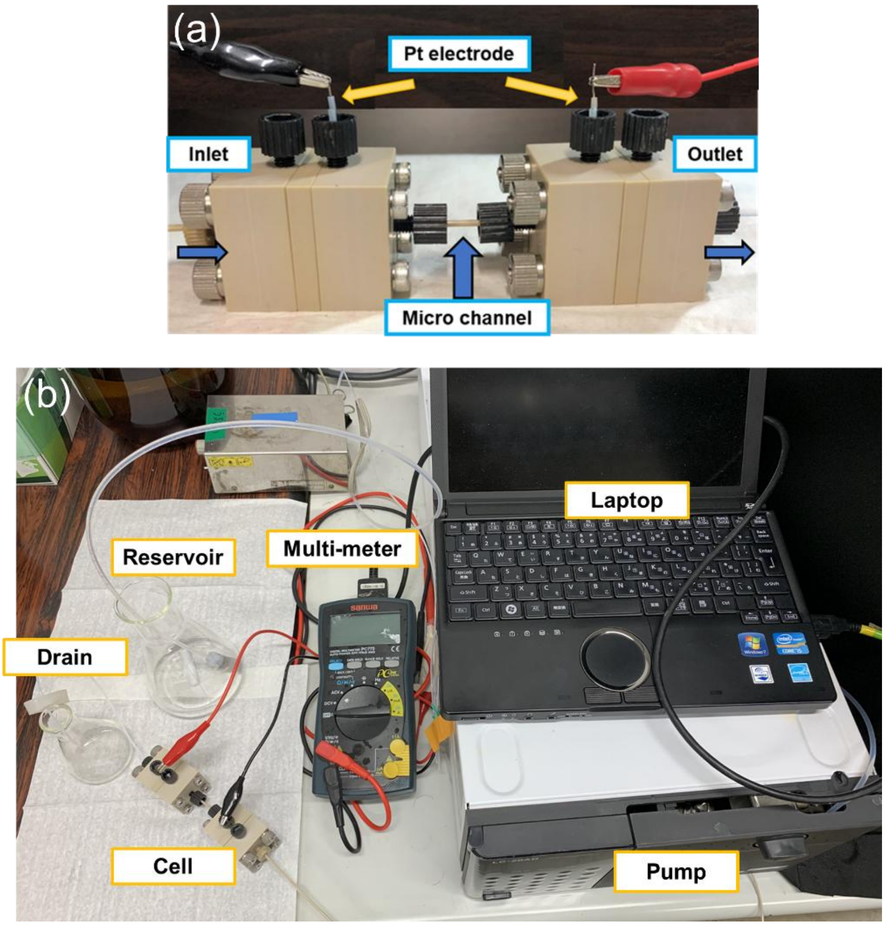

Supplementary Figure 1. (a) Photograph of the PEEK cell chambers composing the streaming potential device. (b) Photograph of the experimental setup composed of a plunger pump, cell units and a multimeter connected to a laptop.

2. Comparison of the two phenolic resin monoliths performances

For convenience of use, two resin monoliths were employed for subsequent experiments and investigations. The two resin monoliths were prepared following the same reported procedure,[1] but they showed slightly different abilities in generating E_{str} values as reported in Supplementary Fig. 2. Nevertheless, they both showed the same trend in ΔP and E_{str} generation, namely ΔP increased linearly with flow rate (Supplementary Fig. 2a) and E_{str} increased linearly with ΔP (Supplementary Fig. 2b), so that both resin monoliths were judged suitable for use. In the following experiments, the type of resin monolith, namely type no. 1 and no. 2, is reported.

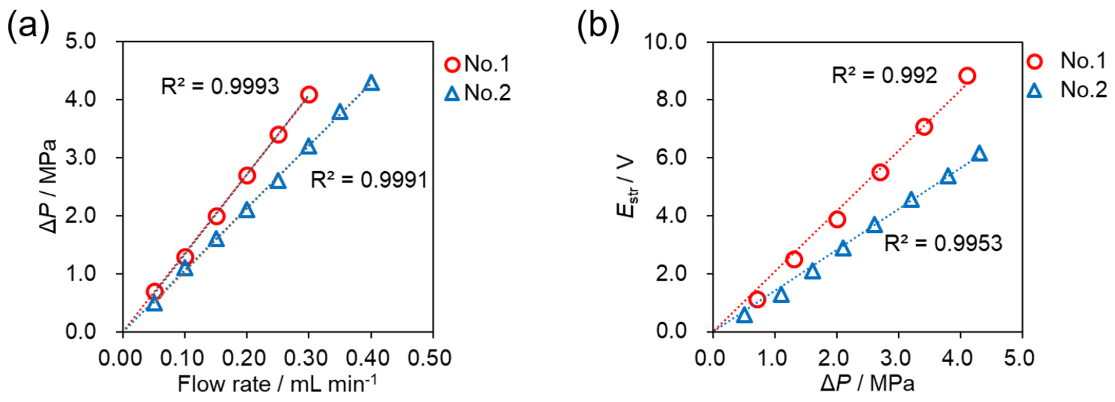

Supplementary Figure 2. Comparison of the two resin monoliths performances by evaluating the relationship between (a) ΔP and flow rate and (b) E_{str} and ΔP.

3. Cell materials for streaming potential measurement

The difference in the cell material had almost no effect on the streaming potential behavior (Supplementary Table 1 and Supplementary Fig. 3).

Supplementary Table 1. Dependence of cell materials on streaming potentials when the MeCN/H_2O (3:1 v/v) solution containing 0.5 mM Bu_4NPF_6 and 5 mM TPrA was pumped using the phenolic resin monolith no. 1 as the filling material.

Entry	Cell material	Flow rate [mL min^{-1}]	ΔP [MPa]	E_{str} [V]
1	PEEK	0.05	0.5	0.94
		0.10	0.9	1.98
		0.15	1.4	3.14
		0.20	1.8	4.35
		0.25	2.2	5.44
		0.30	2.7	6.62
2	Quartz	0.05	0.5	0.89
		0.10	0.9	1.96
		0.15	1.4	3.18
		0.20	1.8	4.31
		0.25	2.2	5.37
		0.30	2.7	6.55

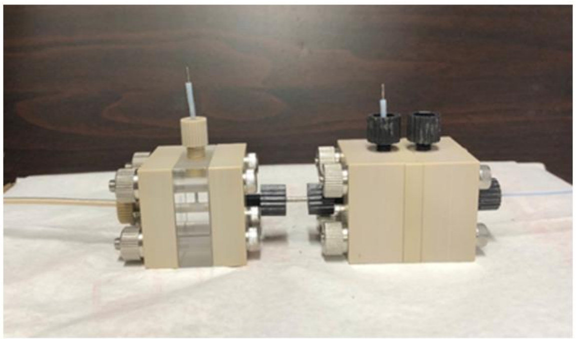

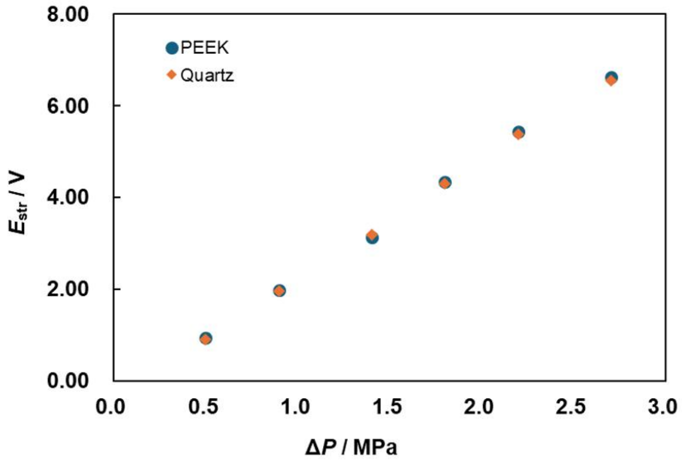

Supplementary Figure 3. Photograph of the streaming potential flow cell equipped with the quartz chamber (upper), and the relationship between E_{str} and ΔP using the PEEK chamber or the quartz chamber (lower). Conditions: TPrA (5 mM) as the coreactant in $MeCN/H_2O$ (3:1 v/v) solution containing 0.5 mM Bu_4NPF_6 as a supporting electrolyte with the phenolic resin monolith no. 1 as the filling material.

4. Formation of BTD-TPA coating onto the electrode

The adopted procedure enabled the formation of a uniform coating of BTD-TPA on the Pt wire (Supplementary Fig. 4) and cyclic voltammetry investigations showed the high reproducibility of the method among different samples (Supplementary Fig. 5).

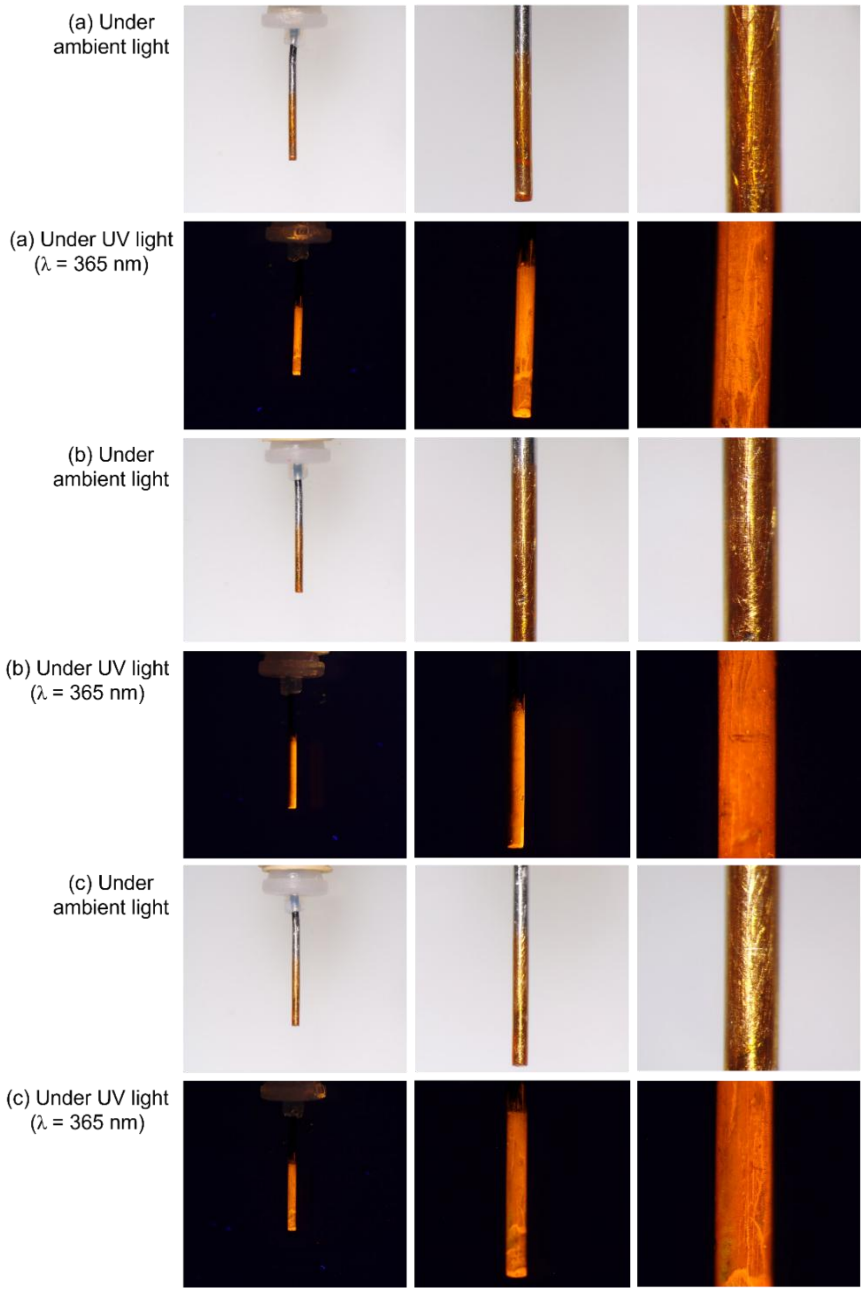

Supplementary Figure 4. Optical images of three different BTD-TPA coatings (samples (a), (b) and (c)) prepared on three different Pt wires under ambient lighting (upper images of each series) and under UV light (excitation wavelength 365 nm) (lower images of each series) at different enlargements. The UV images particularly shows the uniform distribution of each BTD-TPA coating on the Pt wires.

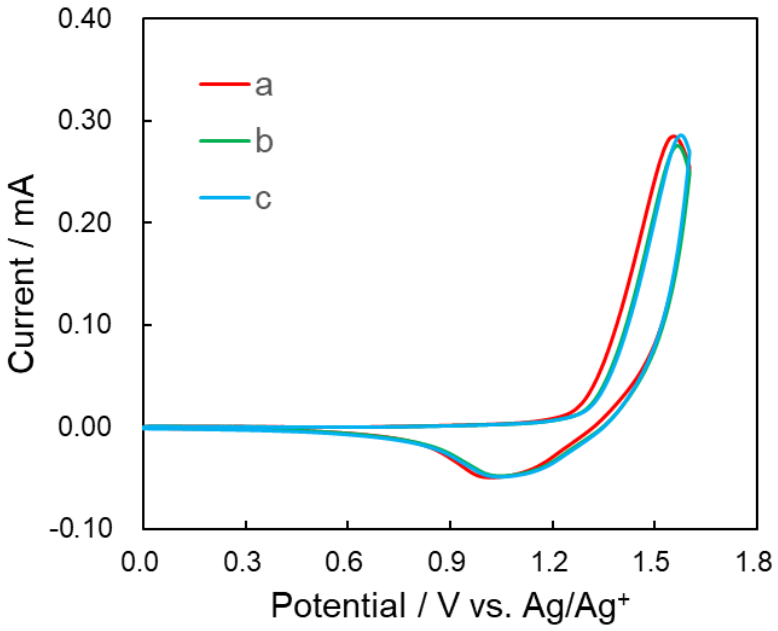

Supplementary Figure 5. Cyclic voltammetry of the three different BTD-TPA coatings prepared on the three different Pt wires shown in Supplementary Fig. 4: sample (a) red line, (b) green line and (c) light blue line. Solution conditions (in air): MeCN/H_2O (3:1 v/v) containing 0.1 M Bu_4NPF_6. Scan rate: 0.1 V s^{-1}.

5. ECL generation and detection in the streaming potential system

A MeCN/H_2O (3:1 v/v) solution containing 0.5 mM Bu_4NPF_6 and 5 mM TPrA was used as the electrolyte and pumped into the cell using a plunger pump. Phenolic resin monolith no. 1 was employed as the filling material. ΔP, E_{str}, and the measured current (i) at each flow rate are summarized in Supplementary Table 2. Supplementary Fig. 6 shows the ECL profiles obtained under the conditions reported in Supplementary Table 2.

Supplementary Table 2. ECL results using TPrA (5 mM) as the coreactant in a MeCN/H_2O (3:1 v/v) solution containing 0.5 mM Bu_4NPF_6. Every set of experiments was repeated three times to check reproducibility.

Entry	Flow rate [mL min^{-1}]	ΔP [MPa]	E_{str} [V]	i [μA]	Integrated ECL intensity [a. u.]
1	0.05	0.7	1.01	0.16	0.004
		0.7	1.04	0.16	
		0.7	1.05	0.16	
2	0.10	1.3	2.32	0.37	0.216
		1.4	2.32	0.37	
		1.4	2.28	0.37	
3	0.15	1.9	3.61	0.57	1.416
		2.0	3.67	0.59	
		2.0	3.58	0.58	
4	0.20	2.7	5.51	0.88	1.522
		2.6	5.18	0.81	
		2.6	5.23	0.82	
5	0.25	3.2	6.71	1.05	1.757
		3.1	6.52	1.03	
		3.2	6.71	1.05	
6	0.30	3.7	8.21	1.27	3.070
		3.7	8.17	1.27	
		3.7	8.05	1.26	

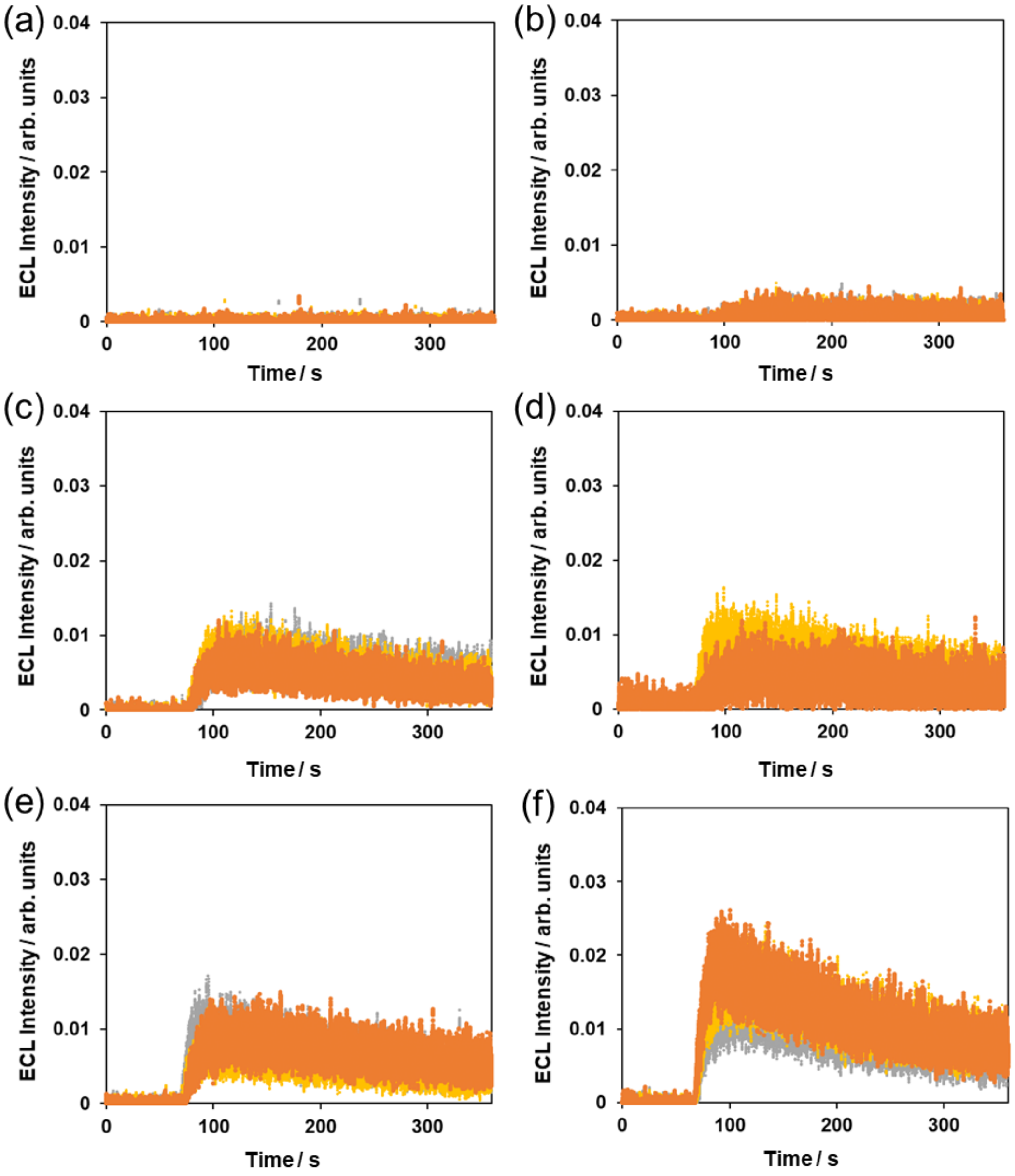

Supplementary Figure 6. ECL intensity of the BTD-TPA film in air during the pumping of the electrolyte for 360 s with the streaming potential device measured under the conditions of (a) Entry 1, (b) Entry 2, (c) Entry 3, (d) Entry 4, (e) Entry 5, (f) Entry 6 of Supplementary Table 2. The experiments were repeated three times (orange, yellow and gray lines) for demonstrating reproducibility. PMT bias = 1000 V.

6. Cyclic voltammetry and ECL measurements with a two-electrode system

To estimate the threshold potential difference for the ECL reaction at the Pt electrode, cyclic voltammetry measurements with a two-electrode system were carried out (Supplementary Fig. 7). The threshold potential difference was estimated to be slightly larger than 0.8 V for both cyclic voltammetry and ECL of a BTD-TPA film in the presence of 5 mM TPrA.

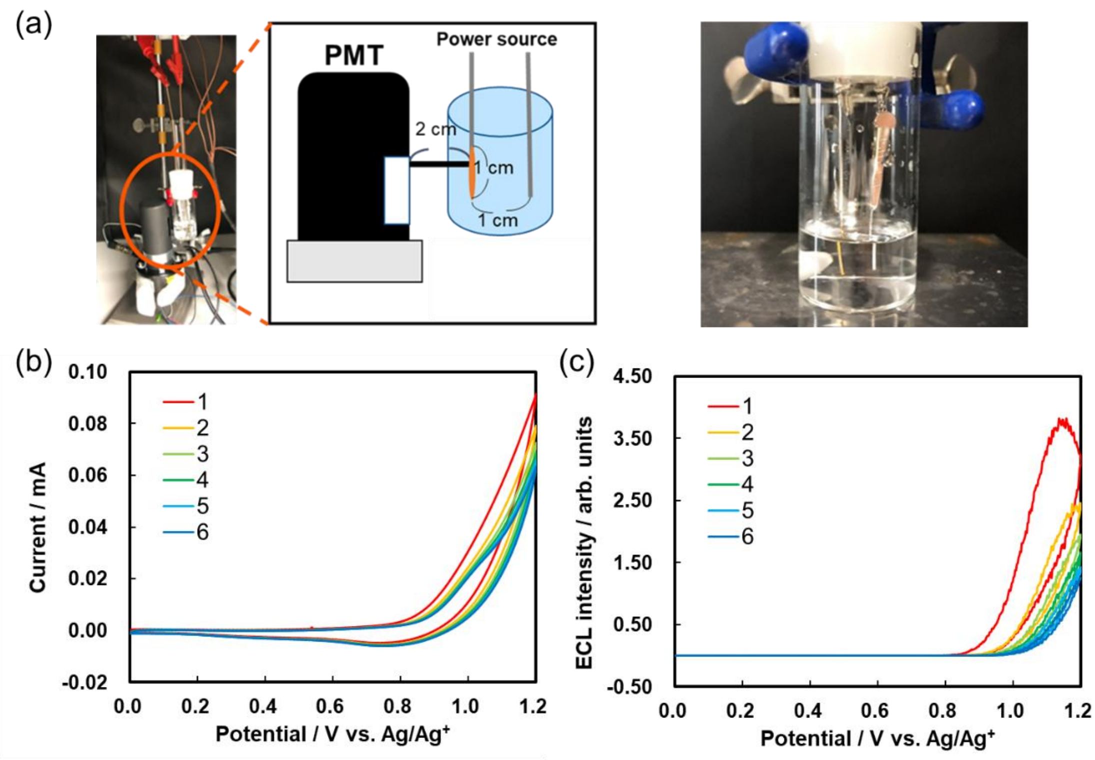

Supplementary Figure 7. (a) Photographs and illustration of the ECL measurement setup of a BTD-TPA film with the two-electrode system (Pt) using the PMT. (b) Current-voltage curves and (c) ECL intensity-voltage curves for a BTD-TPA film in air during the voltage sweep for 6 cycles with the two-electrode system in a mixed solution of MeCN and H_2O (3:1 v/v) containing 0.5 mM Bu_4NPF_6 and 5 mM TPrA. PMT bias = 1000 V.

7. Optimization of the amount of BTD-TPA coating onto the anode

The streaming potential ECL device was driven by the flow of a MeCN/H_2O (3:1 v/v) solution containing 0.5 mM Bu_4NPF_6 and 5 mM TPrA. To optimize the amount of BTD-TPA coating onto the electrode, the investigation of the dip-coating films obtained from chloroform solutions with different concentration of BTD-TPA was carried out. The ECL results are summarized in Supplementary Table 3 and Supplementary Fig. 8. Phenolic resin monolith no. 1 was employed as the filling material.

Supplementary Table 3. Dependence of the ECL intensity on the BTD-TPA coating onto the electrode. Conditions: TPrA (5 mM) as the coreactant in a MeCN/H_2O (3:1 v/v) solution containing 0.5 mM Bu_4NPF_6 as supporting electrolyte.

Entry	Concentration of BTD-TPA [mg mL^{-1}]	Amount of coating [μg mm^{-2}]	Flow rate [mL min^{-1}]	ΔP [MPa]	E_{str} [V]	i [μA]	Integrated ECL intensity [a. u.]
1	10	0.26	0.05	0.6	0.96	0.15	0
			0.10	1.2	2.09	0.36	0
			0.15	1.8	3.32	0.57	0.070
			0.20	2.5	4.60	0.79	0.103
			0.25	3.0	5.93	1.01	0.090
			0.30	3.6	6.836	1.14	0.220
2	20	0.71	0.05	0.7	1.03	0.16	0.003
			0.10	1.4	2.30	0.37	0.211
			0.15	2.0	3.59	0.58	1.400
			0.20	2.6	5.20	0.82	1.522
			0.25	3.2	6.71	1.05	1.909
			0.30	3.7	8.19	1.27	2.892
3	30	0.90	0.05	0.8	0.90	0.16	0
			0.10	1.6	2.08	0.37	0
			0.15	2.3	3.41	0.60	0.354
			0.20	3.0	4.83	0.83	0.662
			0.25	3.6	6.23	1.05	1.252
			0.30	4.2	7.72	1.29	3.183

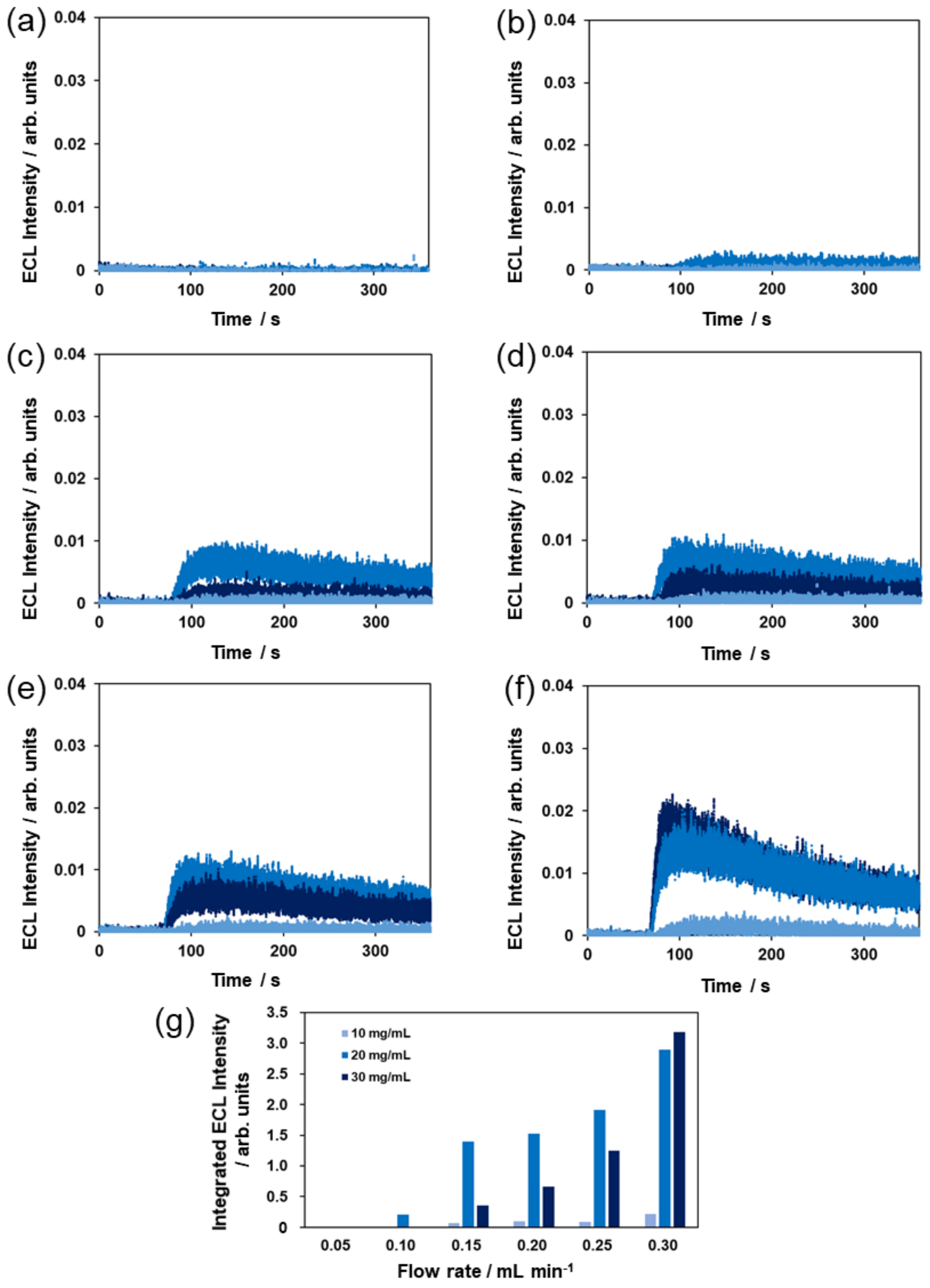

Supplementary Figure 8. ECL intensity of the different BTD-TPA films in air during the pumping of the electrolyte for 360 s with the streaming potential device measured at the different flow rates of (a) 0.05 mL min^{-1}, (b) 0.10 mL min^{-1}, (c) 0.15 mL min^{-1}, (d) 0.20 mL min^{-1}, (e) 0.25 mL min^{-1}, (f) 0.30 mL min^{-1} as reported in Supplementary Table 3. (g) Integrated ECL intensity for each concentration of BTD-TPA coating solution. PMT bias = 1000 V.

8. Optimization of solvent composition

To optimize the solvent composition of the streaming potential ECL device, different solvent ratios of MeCN and H_2O were investigated using the optimized amount of BTD-TPA coating onto the electrode (0.71 μg mm^{-2}) and keeping the concentrations of supporting electrolyte and coreactant constant (0.5 mM Bu_4NPF_6 and 5 mM TPrA, respectively). Phenolic resin monolith no. 1 was employed as the filling material. The results are summarized in Supplementary Table 4 and Supplementary Fig. 9.

Supplementary Table 4. Dependence of the ECL intensity on the solvent composition. Conditions: 5 mM TPrA as the coreactant in mixed MeCN/H_2O solutions containing 0.5 mM Bu_4NPF_6 as supporting electrolyte.

Entry	Solvent MeCN/H_2O (v/v)	Flow rate [mL min^{-1}]	ΔP [MPa]	E_{str} [V]	i [μA]	Maximum ECL intensity [a. u.]	Integrated ECL intensity [a. u.]
1	2 : 1	0.05	0.8	1.07	0.17	0.0022	0
		0.10	1.5	2.42	0.39	0.0016	0.021
		0.15	2.2	3.64	0.59	0.0025	0.070
		0.20	3.0	5.25	0.84	0.0068	0.270
		0.25	3.8	6.98	1.08	0.0038	0.281
		0.30	4.5	7.55	1.26	0.0041	0.337
2	3 : 1	0.05	0.7	1.03	0.16	0.0017	0.003
		0.10	1.4	2.30	0.37	0.0031	0.211
		0.15	2.0	3.59	0.58	0.0099	1.400
		0.20	2.6	5.20	0.82	0.0109	1.522
		0.25	3.2	6.71	1.05	0.0130	1.909
		0.30	3.7	8.19	1.27	0.0201	2.892
3	4 : 1	0.05	0.6	0.76	0.13	0.0016	0
		0.10	1.2	1.80	0.32	0.0027	0.136
		0.15	1.8	2.98	0.52	0.0049	0.413
		0.20	2.4	4.30	0.73	0.0080	0.852
		0.25	2.9	5.28	0.92	0.0093	1.273
		0.30	3.5	6.70	1.13	0.0196	2.818

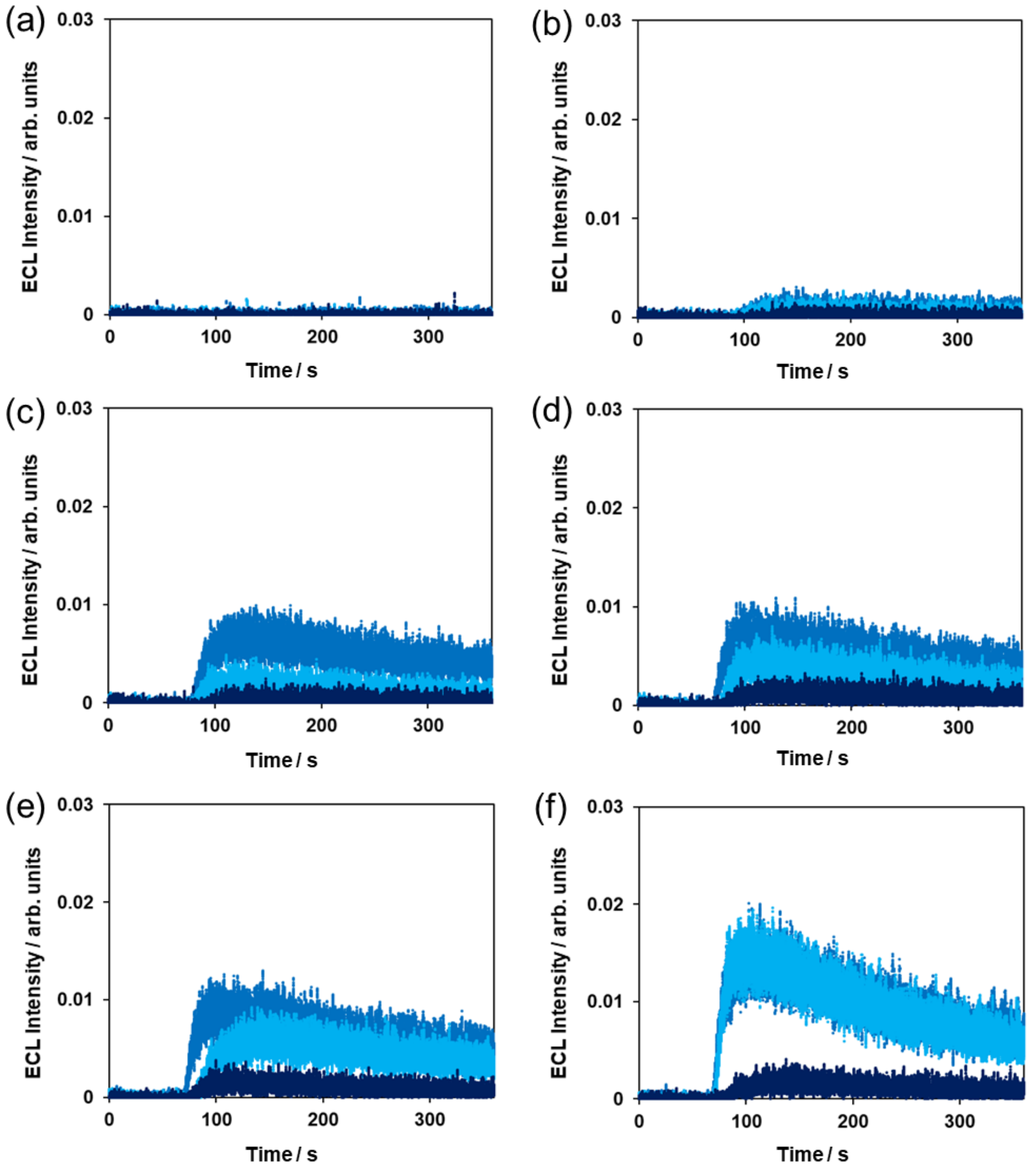

Supplementary Figure 9. ECL intensity of the BTD-TPA film in air during the pumping of the different solvent compositions for 360 s with the streaming potential device measured at the different flow rates of (a) 0.05 mL min^{-1}, (b) 0.10 mL min^{-1}, (c) 0.15 mL min^{-1}, (d) 0.20 mL min^{-1}, (e) 0.25 mL min^{-1}, (f) 0.30 mL min^{-1} as reported in Supplementary Table 4. MeCN/H_2O = 2:1 (deep blue), 3:1 (blue), 4:1 (sky blue). PMT bias = 1000 V.

9. Electrolyte-free condition

To investigate the effect of the supporting electrolyte, the streaming potential ECL device was driven by flowing a MeCN/H_2O (3:1 v/v) solution containing only 5 mM of TPrA. Phenolic resin monolith no. 1 was employed as the filling material. The results are summarized in Supplementary Table 5 and Supplementary Fig. 10.

Supplementary Table 5. Effect of the electrolyte-free *vs.* dilute solution on the ECL measurement intensity. Conditions: TPrA (5 mM) as the coreactant in a MeCN/H_2O (3:1 v/v) solution.

Entry	Electrolyte	Flow rate [mL min^{-1}]	ΔP [MPa]	E_{str} [V]	i [μA]	Maximum ECL intensity [a. u.]	Integrated ECL intensity [a. u.]
1	Free	0.05	0.8	1.33	0.18	0.0019	0.046
		0.10	1.5	2.51	0.38	0.0053	0.581
		0.15	2.2	3.33	0.55	0.0180	2.357
		0.20	3.0	5.70	0.80	0.0313	4.369
		0.25	3.7	7.40	1.03	0.0373	5.362
		0.30	4.4	9.15	1.23	0.0428	6.269
2	Bu_4NPF_6 0.5 mM	0.05	0.7	1.03	0.16	0.0017	0.003
		0.10	1.4	2.30	0.37	0.0031	0.211
		0.15	2.0	3.59	0.58	0.0099	1.400
		0.20	2.6	5.20	0.82	0.0109	1.522
		0.25	3.2	6.71	1.05	0.0130	1.909
		0.30	3.7	8.19	1.27	0.0201	2.892

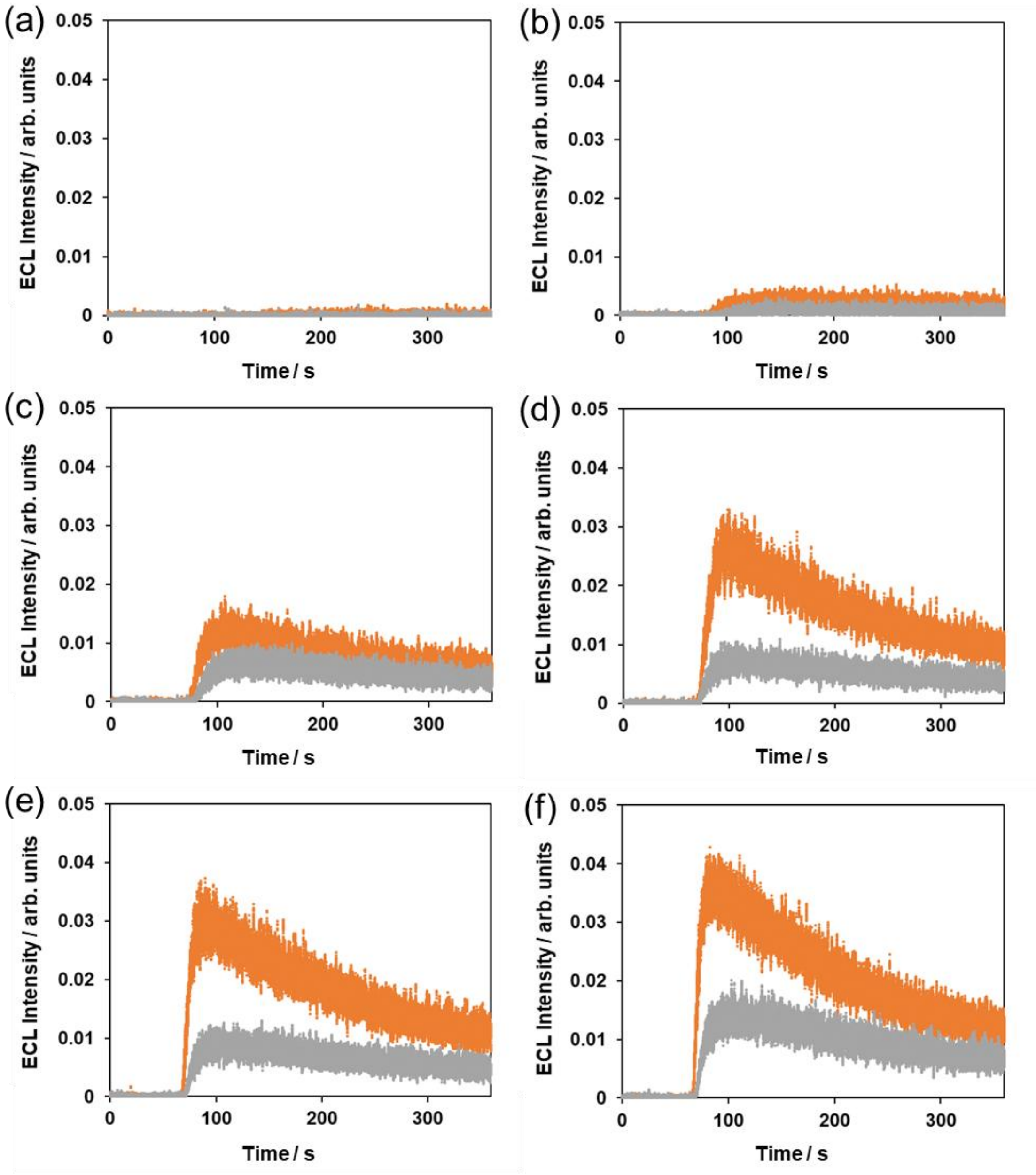

Supplementary Figure 10. ECL intensity of the BTD-TPA film in air during the pumping of the solution for 360 s with the streaming potential device measured at the different flow rate of (a) 0.05 mL min^{-1}, (b) 0.10 mL min^{-1}, (c) 0.15 mL min^{-1}, (d) 0.20 mL min^{-1}, (e) 0.25 mL min^{-1}, (f) 0.30 mL min^{-1} as reported in Supplementary Table 5. Electrolyte-free solution (orange line) and 0.5 mM Bu_4NPF_6 solution (gray line). PMT bias = 1000 V.

10. Optimization of TPrA concentration

To investigate the effect of TPrA concentration on the ECL emission, the streaming potential ECL device was driven by flowing an electrolyte-free MeCN/H_2O solution (3:1 v/v) with different concentrations of TPrA (10, 5.0, 1.0, 0.0 mM). The results are summarized in Supplementary Table 6 and Supplementary Fig. 11.

Supplementary Table 6. Dependence of the ECL intensity on the TPrA concentration in an electrolyte-free MeCN/H_2O (3:1 v/v) solution.

Entry	TPrA concentration [mM]	Flow rate [mL min^{-1}]	ΔP [MPa]	E_{str} [V]	i [μA]	Maximum ECL intensity [a. u.]	Integrated ECL intensity [a. u.]	pH†	Conductivity [mS m^{-1}]
1	10	0.05	0.7	1.46	0.18	0.0010	0	11.30	1.19
		0.10	1.4	2.76	0.38	0.0013	0		
		0.15	2.1	3.59	0.57	0.0018	0.049		
		0.20	2.8	5.42	0.77	0.0045	0.198		
		0.25	3.4	7.06	0.97	0.0050	0.407		
		0.30	4.2	8.79	1.21	0.0149	1.469		
2	5.0	0.05	0.8	1.33	0.18	0.0020	0.046	11.13	1.11
		0.10	1.5	2.51	0.38	0.0053	0.581		
		0.15	2.2	3.33	0.55	0.0180	2.357		
		0.20	3.0	5.70	0.80	0.0313	4.369		
		0.25	3.7	7.40	1.03	0.0373	5.362		
		0.30	4.4	9.15	1.23	0.0428	6.269		
3	1.0	0.05	0.3	0.95	0.11	0.0016	0.026	10.47	0.90
		0.10	0.7	1.89	0.24	0.0106	1.529		
		0.15	1.0	3.00	0.39	0.0279	4.820		
		0.20	1.4	4.21	0.55	0.0757	14.660		
		0.25	1.8	5.60	0.74	0.1983	36.292		
		0.30	2.2	6.82	0.90	0.2670	47.884		
4	0.0	0.05	0.9	1.02	0.11	0.0010	0	N/A	0.11
		0.10	1.7	2.07	0.24	0.0009	0		
		0.15	2.5	3.27	0.38	0.0010	0		
		0.20	3.4	4.77	0.55	0.0013	0		
		0.25	4.1	5.81	0.67	0.0013	0		
		0.30	4.7	6.94	0.86	0.0009	0		

†Measured pH with a conductivity value less than 10 mS m^{-1} is to be considered as a reference value.

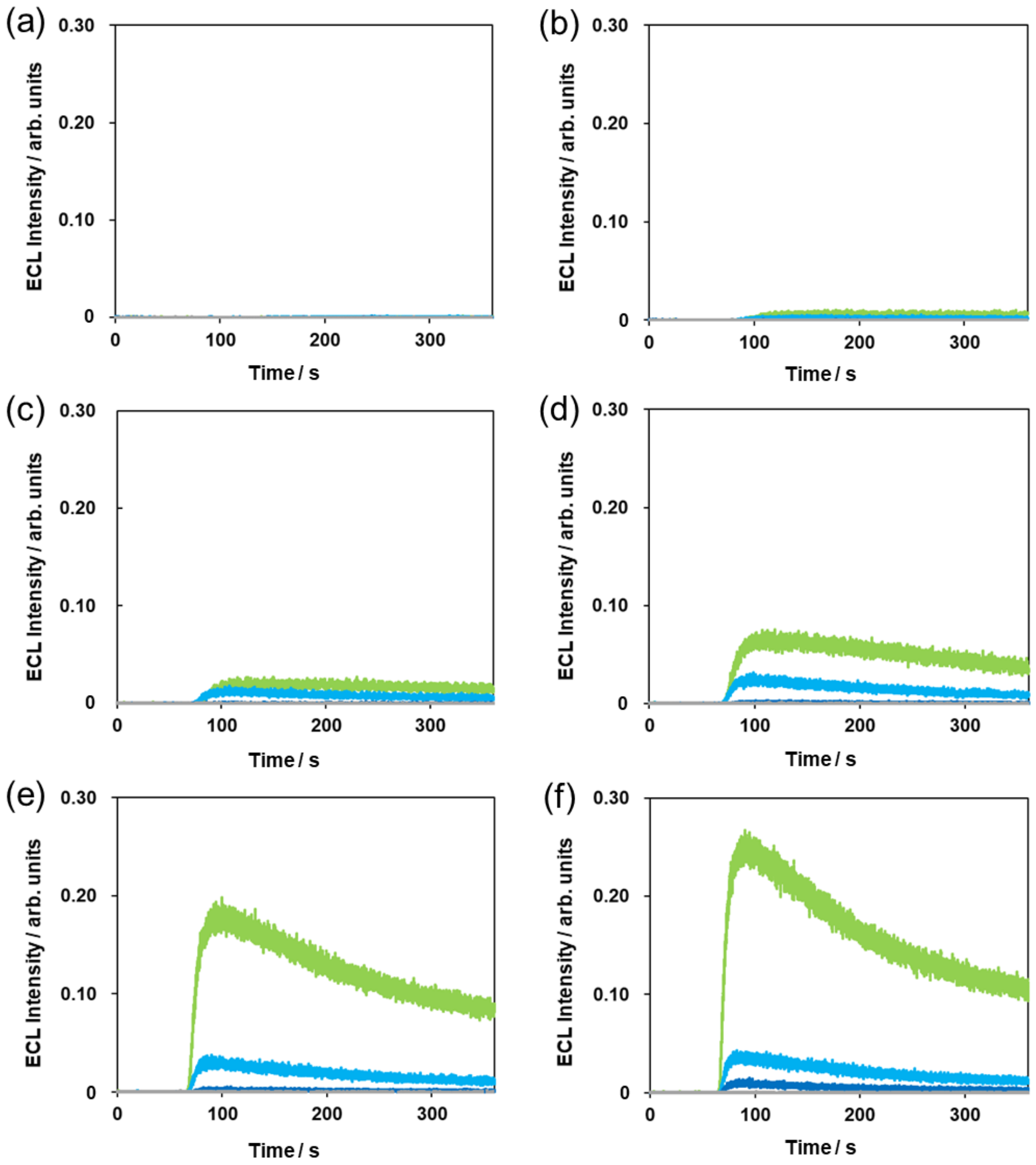

Supplementary Figure 11. ECL intensity of the BTD-TPA film in air during the pumping of the solution for 360 s with the streaming potential device measured at the different flow rate of (a) 0.05 mL min^{-1}, (b) 0.10 mL min^{-1}, (c) 0.15 mL min^{-1}, (d) 0.20 mL min^{-1}, (e) 0.25 mL min^{-1}, (f) 0.30 mL min^{-1} (Supplementary Table 6). The dependence of the ECL emission on TPrA concentration is shown in each curve (gray line: 0.0 mM, green line: 1.0 mM, sky blue line: 5.0 mM, blue line: 10 mM). Phenolic resin monolith no. 1 was employed as the filling material. PMT bias = 1000 V.

11. Impact of the electrode material on the ECL performances of the device

In order to fully evaluate the ECL performances of the device, we also investigated the effect of the electrode (anode) material of the split BPE system. To this purpose, we have replaced the Pt wire acting as the anode with different metals, such as gold (Au), silver (Ag) and copper (Cu), and by dipping each wire in a 20 mg mL^{-1} of BTD-TPA solution and then drying them at the air. All wires used had a constant diameter of 0.6 mm. Phenolic resin monolith no. 2 was employed as the filling material. An electrolyte-free solution composed of MeCN/H_2O (3:1 v/v) containing 1 mM TPrA was flown at 0.3 mL min^{-1}. The results are summarized in Supplementary Table 7 and Supplementary Fig. 12.

Supplementary Table 7. Effect of the electrode material on the ECL generation when flowing an electrolyte-free solution composed of MeCN/H_2O (3:1 v/v) containing 1 mM TPrA at the flow rate of 0.3 mL min^{-1}. All wires had a constant diameter. Cathode material was Pt (ϕ = 0.6 mm) in all cases.

Entry	Material	ΔP [MPa]	E_{str} [V]	i [μA]	Maximum ECL intensity [a. u.]	Integrated ECL intensity [a. u.]
1	Pt	2.1	5.68	0.69	0.30	58.8
2	Au	2.1	5.78	0.69	0.11	25.9
3	Ag	2.1	5.83	0.69	0.00	0.00
4	Cu	2.1	5.88	0.71	0.00	0.00

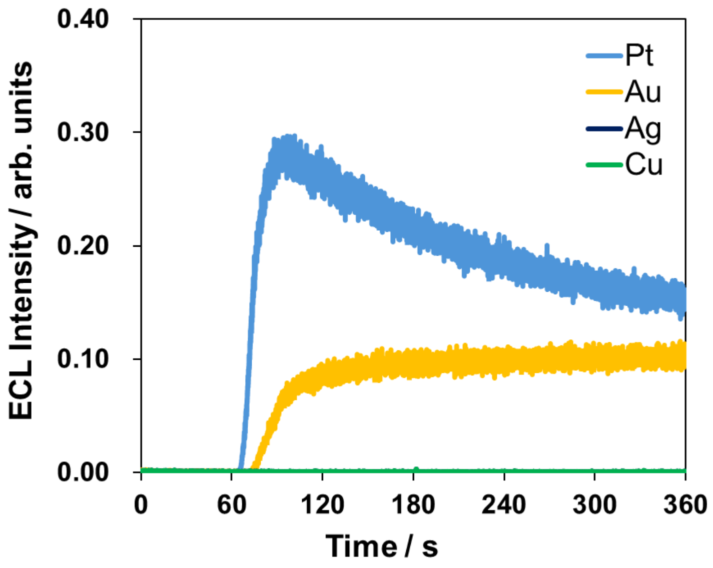

Supplementary Figure 12. ECL intensity of the BTD-TPA film in air during the pumping of the solution for 360 s with the streaming potential device using different electrode materials as the anode constituting the split BPE system: Pt (light blue line), Au (orange line), Ag (dark blue line) and Cu (green line). Conditions: 1 mM TPrA in an electrolyte-free MeCN/H_2O (3:1 v/v) solution flown at the flow rate of 0.3 mL min^{-1}. Phenolic resin monolith no. 2 was employed as the filling material. PMT bias = 1000 V.

12. ECL imaging with a digital camera

Using the optimal conditions of Supplementary Table 6 (1 mM TPrA, electrolyte-free MeCN/H_2O (3:1 v/v), 0.30 mL min^{-1} of flow rate, phenolic resin monolith no. 1 as the filling material), the strong ECL intensity could be recorded by using a PMT. We next explored the possibility to detect the ECL emission by using a simple digital camera as shown in Supplementary Fig. 13a. The camera parameters for still imaging were ISO 25600, f/2.8, exposure time 30 sec; for movie: ISO 12800, 30 fps. The results are summarized in Supplementary Table 8, Supplementary Fig. 13 and Supplementary Movie 1.

Supplementary Table 8. Camera parameters for ECL imaging. Solution conditions: TPrA 1 mM in an electrolyte-free MeCN/H_2O (3:1 v/v) solution.

Entry	Imaging	Flow rate [mL min^{-1}]	ΔP [MPa]	E_{str} [V]	i [μA]	Maximum ECL intensity [a. u.]	Integrated ECL intensity [a. u.]
1	Still	0.30	2.2	6.68	0.90	0.2345	42.835
2	Movie	0.50	3.7	11.50	1.54	0.7853	115.793

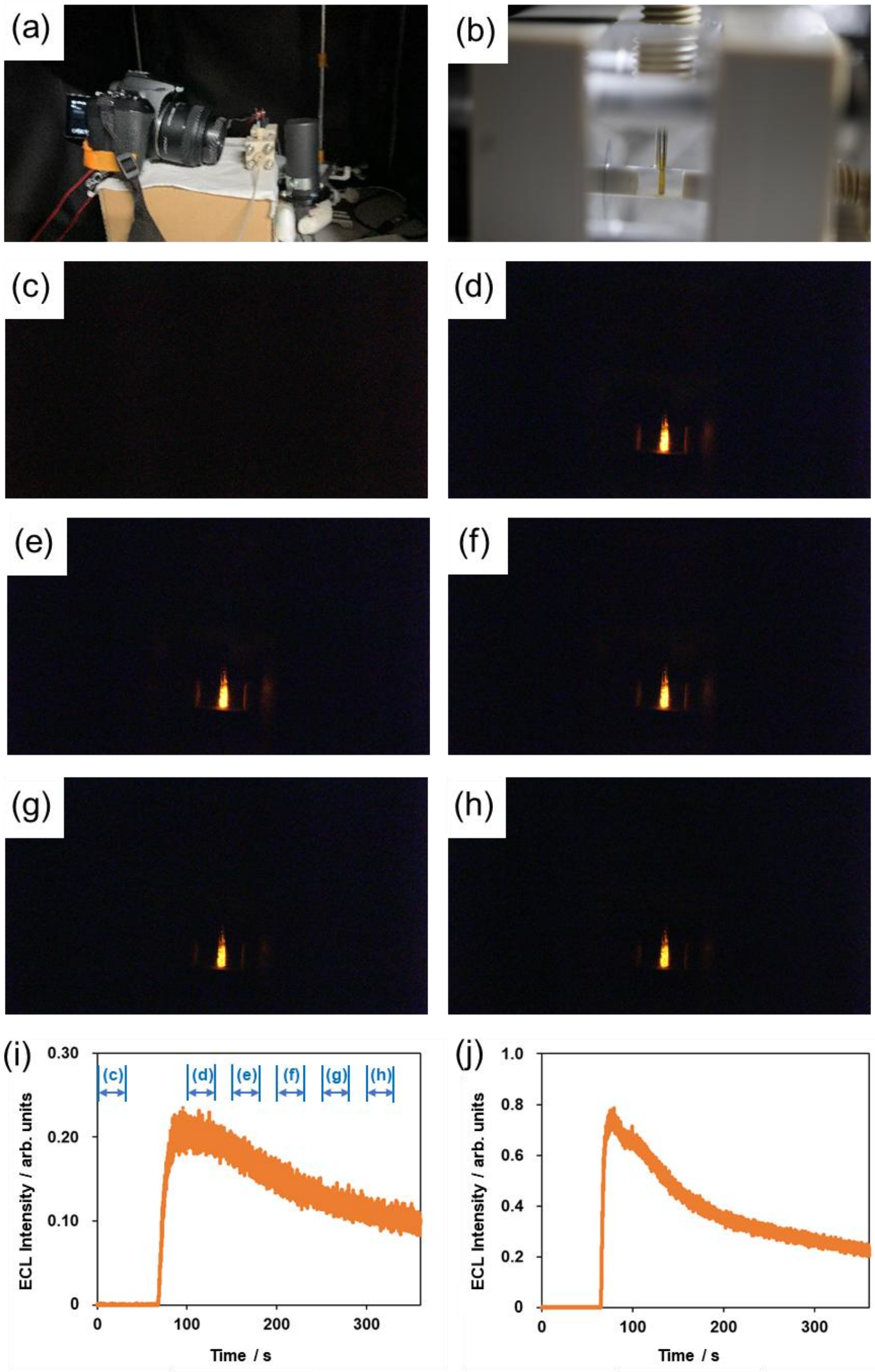

Supplementary Figure 13. (a) Setup for the simultaneous ECL imaging acquisition using a digital camera (located on the left side of the quartz cell) and recording of the ECL intensity profile using a PMT (located on the right). (b) Photograph of the quartz cell under natural light showing the BTD-TPA coated electrode. (c–h) Collected ECL images (exposure time 30 sec each) during the flow of the solution using the conditions of entry 1 in Supplementary Table 8 at (c) 0 sec, (d) 100 sec, (e) 150 sec, (f) 200 sec, (g)

250 sec, and (h) 300 sec of device operation. (i, j) ECL intensity of the BTD-TPA film in air during the pumping of the solution for 360 s with the streaming potential device measured using the conditions of Supplementary Table 8, for (i) entry 1 and (j) entry 2. PMT bias = 1000 V. Camera parameters for still imaging: ISO 25600, f/2.8, exposure time 30 sec. Camera parameters for movie: ISO 12800, 30 fps. The (c) to (h) letters indicated in graph (i) correspond to the location and duration of the camera acquisition applied to record the still images reported in panels (c) to (h).

13. ECL spectra measurement

Using the optimized conditions of Supplementary Table 6 (1 mM TPrA in an electrolyte-free MeCN/H_2O (3:1 v/v), 0.50 mL min^{-1} of flow rate, phenolic resin monolith no. 1 as the filling material), the ECL spectra were collected with a spectrometer by placing the optical fiber cable close to the quartz cell chamber (as shown in Supplementary Fig. 14a). The obtained ECL spectrum of BTD-TPA (Supplementary Fig. 14b) corresponded to that reported in literature by using a three-electrode system.[2,3]

(a)

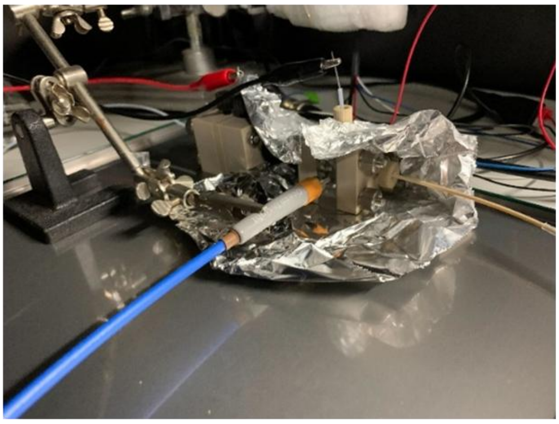

(b)

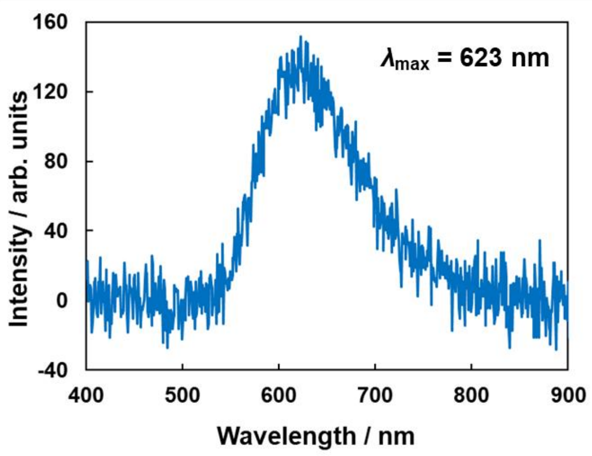

Supplementary Figure 14. (a) Photograph of the apparatus for recording the ECL spectrum of the BTD-TPA film using a spectrometer with an optical fiber cable set close to the quartz cell chamber. (b) ECL spectrum of a BTD-TPA film obtained using the streaming potential device to drive the ECL reaction. Conditions: 1 mM TPrA in an electrolyte-free MeCN/H_2O (3:1 v/v) solution at the flow rate of 0.50 mL min^{-1}.

14. Pulsed flow for the ECL system

To investigate the ECL response respect to the on/off pumping of the solution, the streaming potential ECL device was driven by interval flow of an electrolyte-free MeCN/H_2O (3:1 v/v) solution containing 1 mM TPrA. An on/off pumping scheme of 10 sec flow and 10 sec pause was repeated 15 times. Phenolic resin monolith no. 1 was employed as the filling material. The results are summarized in Supplementary Table 9 and Supplementary Figs. 15 and 16.

Supplementary Table 9. Solution conditions for the on/off pumping scheme: 1 mM TPrA in an electrolyte-free MeCN/H_2O (3:1 v/v) solution.

Entry	Flow rate [mL min^{-1}]	ΔP Max [MPa]	E_{str} Max [V]	i Max [μA]
1	0.10	0.8	2.41	0.29
2	0.20	1.7	5.10	0.60
3	0.30	2.5	8.01	0.90

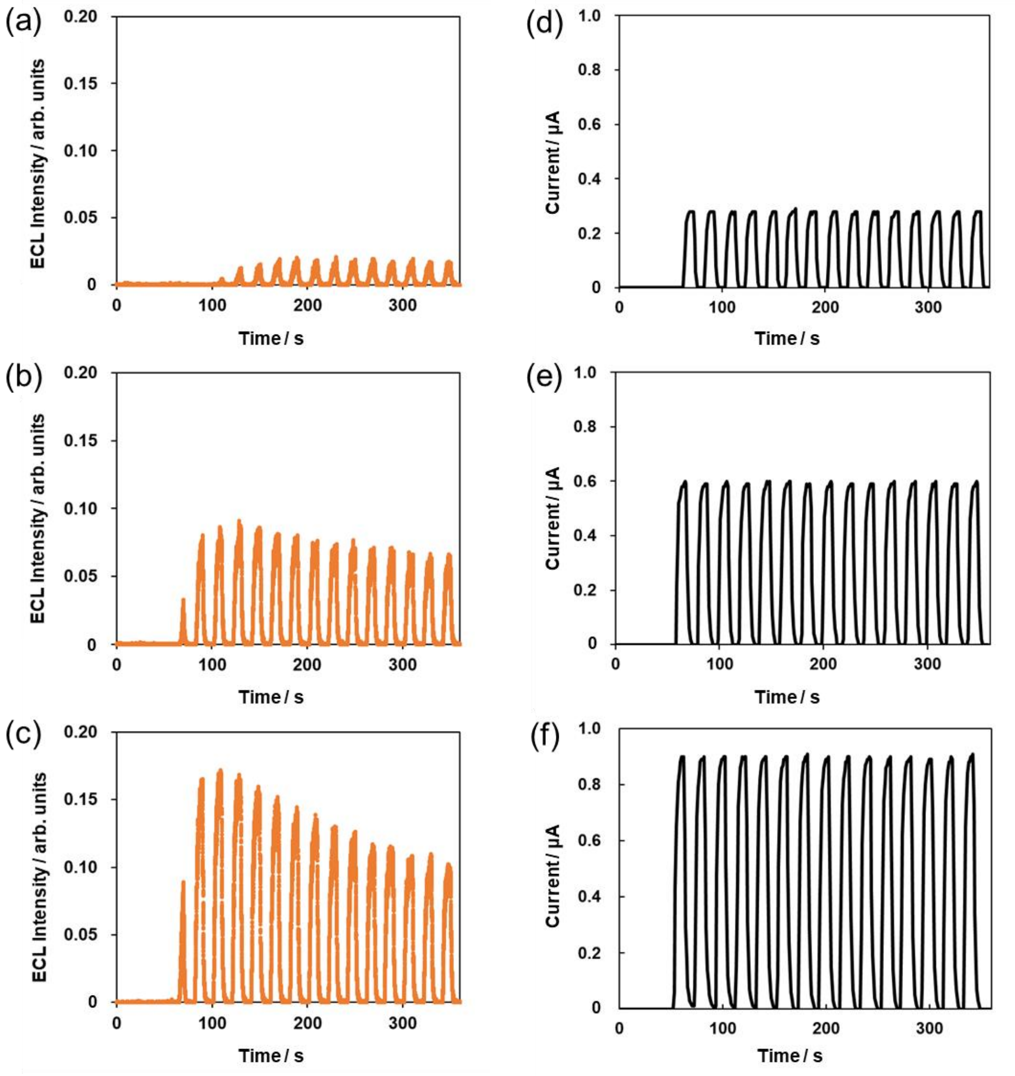

Supplementary Figure 15. ECL intensity (orange line graphs) and current profiles (gray line graphs) recorded at the flow rate of (a, d) 0.10 mL min^{-1}, (b, e) 0.20 mL min^{-1}, (c, f) 0.30 mL min^{-1} as reported in Supplementary Table 8. Conditions: 1 mM TPrA in an electrolyte-free MeCN/H_2O (3:1 v/v) solution. PMT bias = 1000 V.

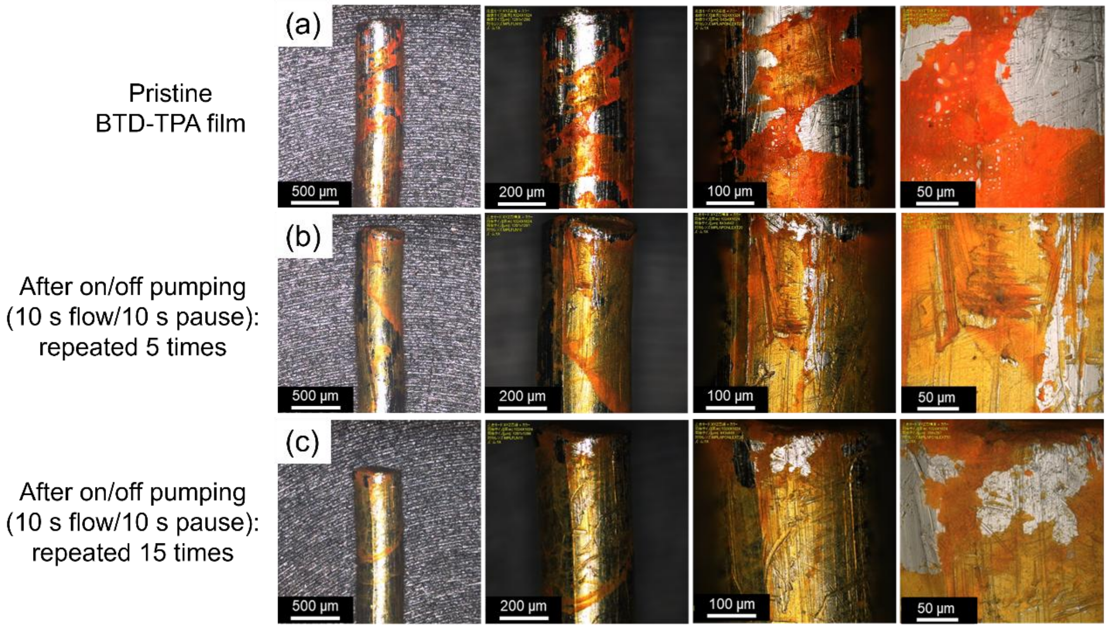

Supplementary Figure 16. 3D laser microscope images of the Pt electrode covered with BTD-TPA film. (a) Pristine BTD-TPA film; BTD-TPA coating after the ECL experiment following the on/off pumping scheme − 10 sec pumping of the solution using the conditions of entry 3 in Supplementary Table 8 and 10 sec stasis of the device − repeated for (b) 5 times and (c) 15 times.

15. Limit of detection (LOD) for TPrA in the device

To evaluate the limit of detection (LOD) for TPrA of the streaming potential ECL device, different electrolyte-free MeCN/H_2O (3:1 v/v) solutions containing 1.0 mM, 0.75 mM, 0.50 mM, 0.25 mM, 0.10 mM, 0.050 mM, 0.010 mM and 0 mM TPrA were flown into the device. Phenolic resin monolith no. 1 was employed as the filling material. The results are summarized in Supplementary Table 10 and Supplementary Fig. 17.

Supplementary Table 10. Dependence of ECL intensity on TPrA concentration (range 1.0–0 mM) in an electrolyte-free MeCN/H_2O (3:1 v/v) solution. Flow rate was 0.30 mL min^{-1} in all cases.

Entry	TPrA concentration [mM]	ΔP [MPa]	E_{str} [V]	i [μA]	Maximum ECL intensity [a. u.]	Integrated ECL intensity [a. u.]
1	1.0	4.2	11.75	1.41	0.1393	45.135
2	0.75	4.4	11.95	1.38	0.3616	70.126
3	0.50	4.2	11.65	1.3	0.4960	91.264
4	0.25	4.2	11.69	1.35	0.3052	61.570
5	0.10	4.3	11.27	1.27	0.0280	3.228
6	0.050	4.1	10.88	1.17	0.0049	0.637
7	0.010	4.5	9.34	1.01	0.0085	0.147
8	0	4.5	5.89	0.61	0	0

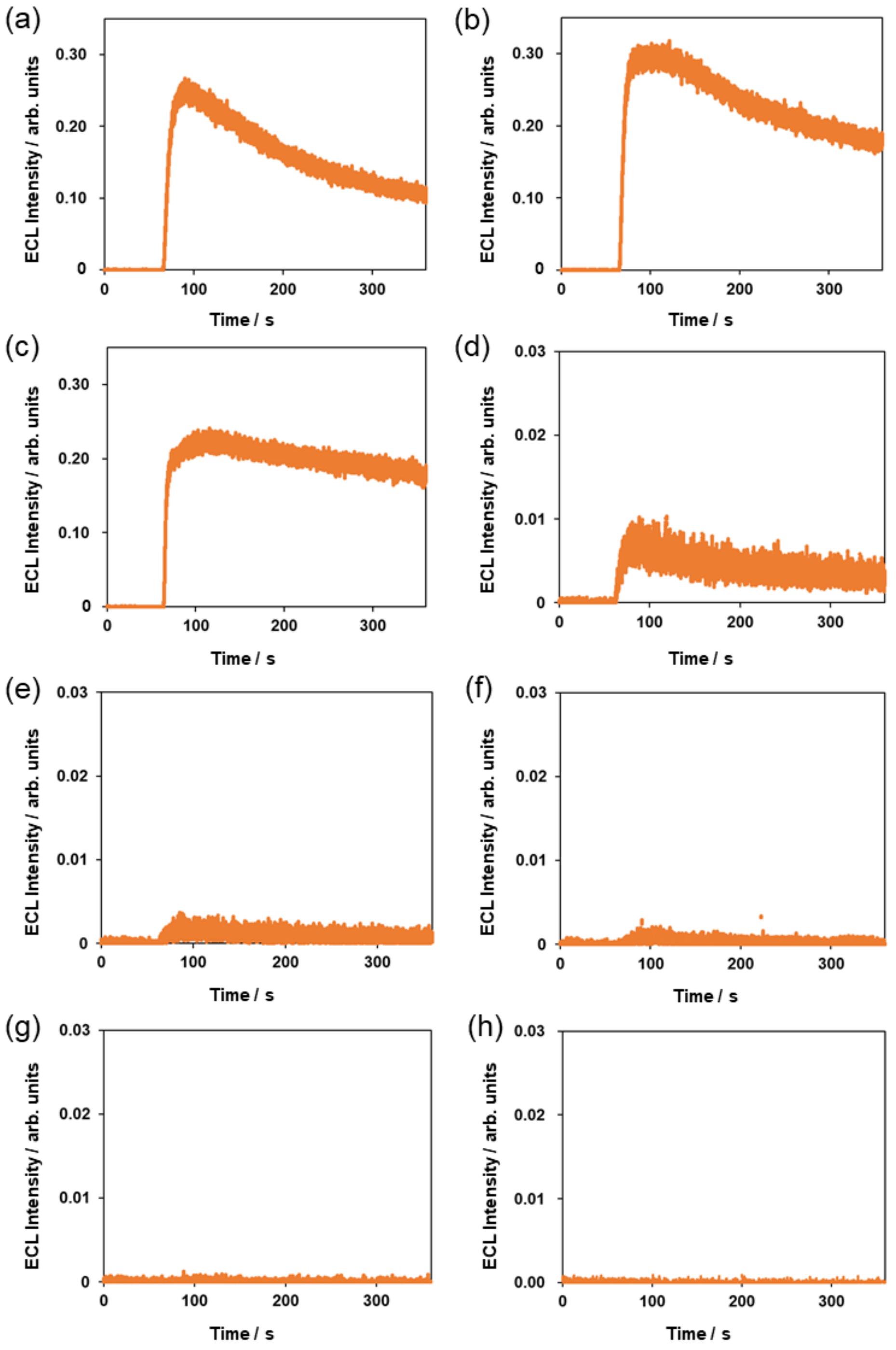

Supplementary Figure 17. ECL intensity of the BTD-TPA film in air during the pumping of the electrolyte for 360 s with the streaming potential device using an electrolyte-free MeCN/H_2O (3:1 v/v) solution with (a) 1.0 mM, (b) 0.50 mM, (c) 0.25 mM, (d) 0.10 mM, (e) 0.05 mM, (f) 0.025 mM, (g) 0.010 mM, (h) 0.0 mM TPrA. PMT bias = 1000 V. Other parameters are summarized in Supplementary Table 10.

16. Detection of other amines

In addition to TPrA as coreactant, other amines such as 2-(dibutylamino)ethanol (DBAE) and triethanolamine (TEOA) were investigated as coreactants for the streaming potential ECL device. The results are summarized in Supplementary Table 11 and Supplementary Fig. 18.

Supplementary Table 11. ECL intensity using different coreactant (1 mM each) in an electrolyte-free MeCN/H_2O (3:1 v/v) solution. Phenolic resin monolith no. 1 was employed as the filling material.

Entry	Coreactant 1 mM	Flow rate [mL min^{-1}]	ΔP [MPa]	E_{str} [V]	i [μA]	Maximum ECL intensity [a. u.]	Integrated ECL intensity [a. u.]	pH†	Conductivity [mS m^{-1}]
1	TPrA	0.05	0.3	0.95	0.11	0.0016	0.026	10.47	0.90
		0.10	0.7	1.89	0.24	0.0106	1.529		
		0.15	1.0	3.00	0.39	0.0279	4.820		
		0.20	1.4	4.21	0.55	0.0757	14.660		
		0.25	1.8	5.60	0.74	0.1983	36.292		
		0.30	2.2	6.82	0.90	0.2670	47.884		
2	DBAE	0.05	0.5	1.10	0.13	0.0052	0.510	9.21	1.05
		0.10	0.9	2.44	0.29	0.0208	3.254		
		0.15	1.3	3.81	0.45	0.0511	8.882		
		0.20	1.7	5.09	0.60	0.0889	16.229		
		0.25	2.1	6.49	0.77	0.0984	17.998		
		0.30	2.5	7.77	0.91	0.1543	25.824		
3	TEOA	0.05	0.4	1.29	0.13	0.0009	0	8.58	0.41
		0.10	0.9	2.64	0.29	0.0009	0		
		0.15	1.3	3.95	0.43	0.0011	0		
		0.20	1.7	5.25	0.57	0.0016	0.029		
		0.25	2.2	6.67	0.72	0.0031	0.061		
		0.30	2.6	8.24	0.92	0.0025	0.122		

†Measured pH with a conductivity value less than 10 mS m^{-1} is to be considered as a reference value.

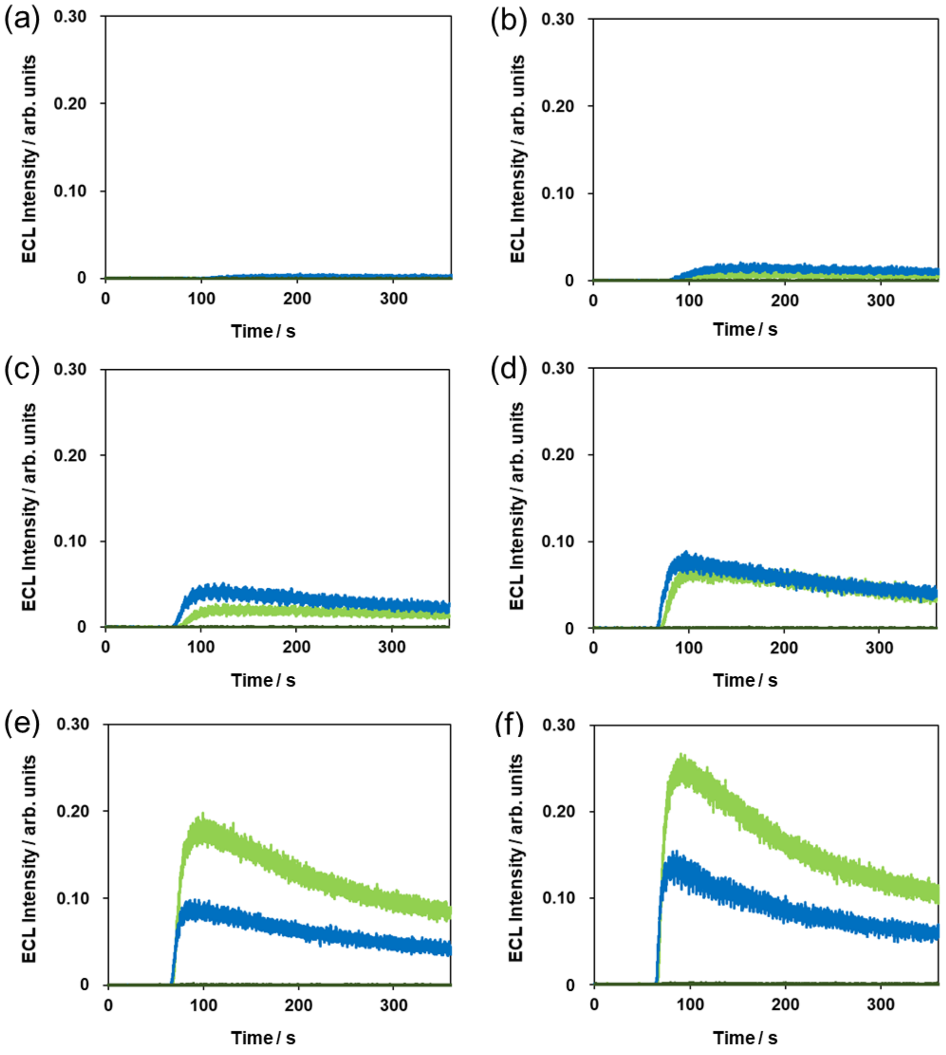

Supplementary Figure 18. ECL intensity of the BTD-TPA film in air during the pumping of the electrolyte for 360 s with the streaming potential device using an electrolyte-free MeCN/H_2O (3:1 v/v) solution at the flow rate of (a) 0.05 mL min^{-1}, (b) 0.10 mL min^{-1}, (c) 0.15 mL min^{-1}, (d) 0.20 mL min^{-1}, (e) 0.25 mL min^{-1}, (f) 0.30 mL min^{-1}, in presence of TPrA (light green line), DBAE (blue line) and TEOA (green line). PMT bias = 1000 V. Other parameters are summarized in Supplementary Table 11.

17. Detection of aromatic amines

Considering the environmental relevance and human hazard of aromatic amines, the device was also tested for the ECL detection of this class of amines, including aniline, *N*-methylaniline and *N,N*-dimethylaniline. The results are summarized in Supplementary Table 12.

Supplementary Table 12. ECL intensity using different aromatic amines as coreactants (1 mM each) solubilized in an electrolyte-free MeCN/H_2O (3:1 v/v) solution at the flow rate of 0.30 mL min^{-1}. Phenolic resin monolith no. 2 was employed as the filling material. PMT bias = 1000 V.

Entry	Coreactant 1 mM	ΔP [MPa]	E_{str} [V]	i [μA]	Maximum ECL intensity [a. u.]	Integrated ECL intensity [a. u.]	pH†	Conductivity [mS m^{-1}]
1	Aniline	2.8	4.90	0.49	0	0	8.83	0.10
2	*N*-methylaniline	2.8	4.74	0.47	0	0	9.21	0.13
3	*N,N*-dimethylaniline	2.9	4.89	0.49	0	0	8.93	0.16

†Measured pH with a conductivity value less than 10 mS m^{-1} is to be considered as a reference value.

18. Aqueous systems (distilled water)

For a more challenging investigation, a fully aqueous system was used as the solvent for the streaming potential ECL device. 1 mM TPrA, DBAE and TEOA were solubilized in distilled water. The results are summarized in Supplementary Table 13 and Supplementary Fig. 19.

Supplementary Table 13. ECL intensity using different coreactant (1 mM each) solubilized in distilled water and without supporting electrolyte at the flow rate of 0.30 mL min^{-1}. Phenolic resin monolith no. 1 was employed as the filling material.

Entry	Coreactant 1 mM	ΔP [MPa]	E_{str} [V]	i [μA]	Maximum ECL intensity [a. u.]	Integrated ECL intensity [a. u.]	pH†	pK_a	Conductivity [mS m^{-1}]
1	TPrA	3.8	11.96	1.38	0.0058	0.274	10.59	10.4[4]	8.49
2	DBAE	4.2	9.71	1.32	0.0041	0.439	10.27	9.9[5]	4.40
3	TEOA	3.7	12.39	1.40	0.0040	0.010	8.85	7.8[6]	0.57

†Measured pH with a conductivity value less than 10 mS m^{-1} is to be considered as a reference value (nevertheless, reflecting the trend of reported pK_a values).

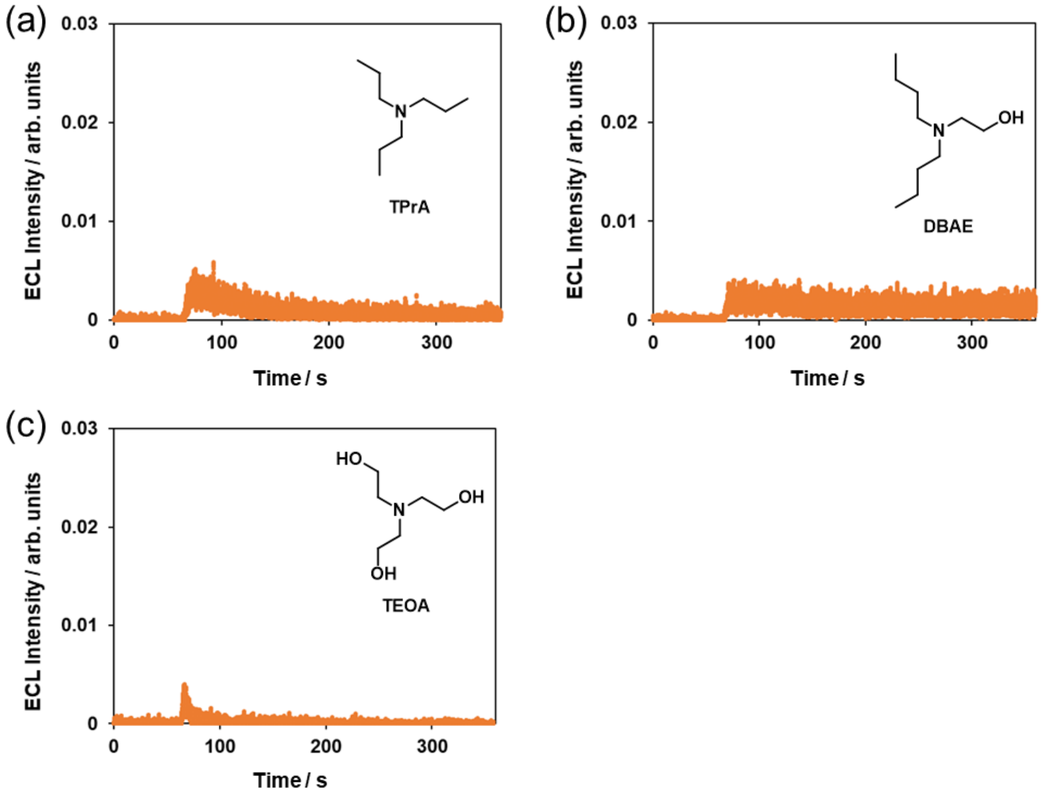

Supplementary Figure 19. ECL intensity for the BTD-TPA film in air during the pumping of the solution for 360 s with the streaming potential device using distilled water without electrolyte as the solvent at the flow rate of 0.30 mL min^{-1}, in presence of (a) 1 mM TPrA, (b) 1 mM DBAE, and (c) 1 mM TEOA. PMT bias = 1000 V.

19. Aqueous systems (tap water)

Tap water[7] was also tested as solvent for the streaming potential ECL device. The results are summarized in Supplementary Table 14 and Supplementary Fig. 20.

Supplementary Table 14. ECL intensity using tap water as the solvent with and without TPrA (1 mM) at the flow rate of 0.30 mL min^{-1}. Phenolic resin monolith no. 1 was employed as the filling material.

Entry	Coreactant 1 mM	ΔP [MPa]	E_{str} [V]	i [μA]	Maximum ECL intensity [a. u.]	Integrated ECL intensity [a. u.]	pH	Conductivity [mS m^{-1}]
1	TPrA	3.9	2.14	0.57	0.0136	0.274	9.57	31.7
2	–	4.1	1.65	0.44	0	0	7.35	30.7

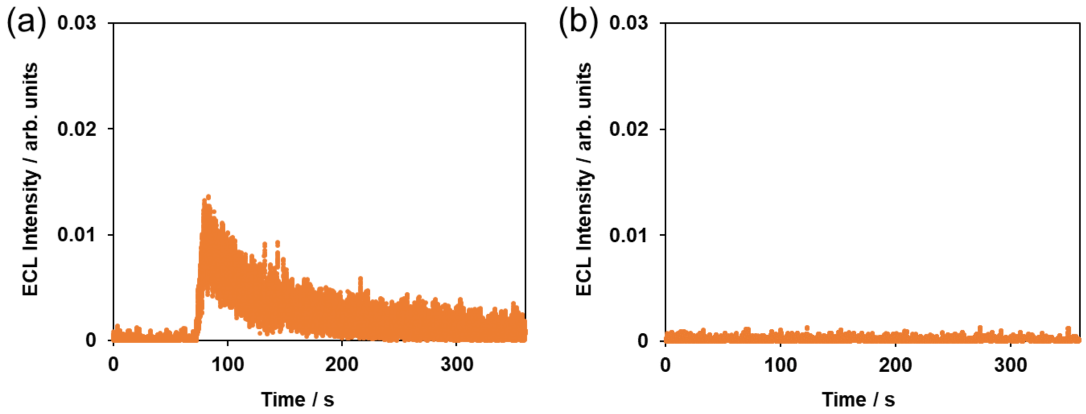

Supplementary Figure 20. ECL intensity for the BTD-TPA film in air during the pumping of the solution for 360 s with the streaming potential device using tap water without electrolyte at the flow rate of 0.30 mL min^{-1} (a) with and (b) without TPrA (1 mM). PMT bias = 1000 V.

20. Evaluation of the anti-interference capabilities of the device

The anti-interference performances of the streaming potential device were additionally investigated in both the standard electrolyte, composed of MeCN/H_2O (3:1 v/v) without supporting electrolyte, and tap water. To this purpose, the ECL detection of multiple amines solubilized in the same solution sample, or with the addition of other analytes, e.g. salt, was carried out using the device. The results are summarized in Supplementary Tables 15 and 16 and Supplementary Figs. 21 and 22.

Supplementary Table 15. Anti-interference performances study based on the ECL detection of multiple amines (coreactants) solubilized in the same solution sample composed of an electrolyte-free MeCN/H_2O (3:1 v/v) solution at the flow rate of 0.30 mL min^{-1}. Phenolic resin monolith no. 2 was employed as the filling material.

Entry	Multiple amines system (coreactants)	ΔP [MPa]	E_{str} [V]	i [μA]	Maximum ECL intensity [a. u.]	Integrated ECL intensity [a. u.]	pH†	Conductivity [mS m^{-1}]
1	1 mM TPrA	2.5	6.41	0.73	0.093	16.24	9.62	3.96
2	1 mM TPrA + 1 mM DBAE	2.5	6.29	0.73	0.030	4.28	9.87	3.62
3	1 mM TPrA + 1 mM TEOA	2.7	6.36	0.72	0.005	0.59	9.77	3.17
4	1 mM TPrA + 1 mM Aniline	2.7	6.17	0.73	0	0	9.59	3.06
5	0.5 mM TPrA + 0.5 mM DBAE	2.8	6.13	0.72	0.068	12.19	9.44	2.95
6	0.25 mM TPrA + 0.25 mM DBAE	3.1	6.48	0.72	0.290	48.46	9.34	1.92
7	2 mM TPrA	3.2	6.24	0.72	0.028	3.73	10.58	1.91

†Measured pH with a conductivity value less than 10 mS m^{-1} is to be considered as a reference value.

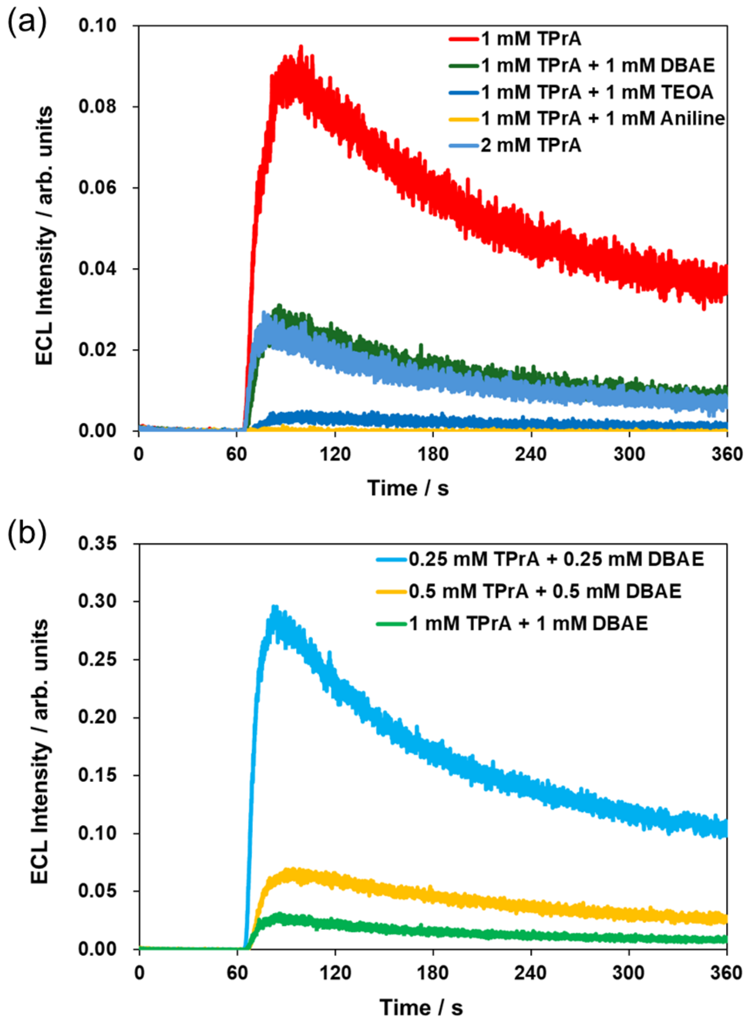

Supplementary Figure 21. ECL intensity of the BTD-TPA film in air during the pumping of the solution for 360 s with the streaming potential device using an electrolyte-free MeCN/H_2O (3:1 v/v) solution at the flow rate of 0.30 mL min^{-1} having a coreactant system composed of (a) the coexistence of TPrA with other aliphatic or aromatic amines and (b) TPrA and DBAE in different concentration ratios. Phenolic resin monolith no. 2 was employed as the filling material. PMT bias = 1000 V.

Supplementary Table 16. Anti-interference performances study based on the ECL detection of 1 mM TPrA solubilized in only tap water and in tap water containing 3% NaCl (0.5 M) at the flow rate of 1.0 mL min^{-1}. The addition of salt in tap water simulates water with high salinity similar to that of sea water. Phenolic resin monolith no. 2 was employed as the filling material.

Entry	Solution system (tap water)	ΔP [MPa]	E_{str} [V]	i [μA]	Maximum ECL intensity [a. u.]	Integrated ECL intensity [a. u.]	pH	Conductivity [mS m^{-1}]
1	1 mM TPrA	4.3	1.54	0.54	0.020	2.57	8.6	33
2	1 mM TPrA + 3% NaCl (0.5 M)	4.6	0	0	0	0	8.9	4.5×10^3

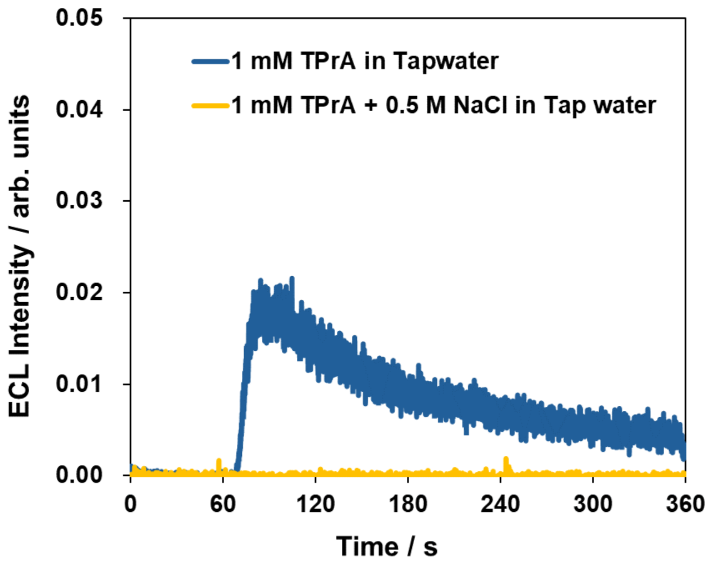

Supplementary Figure 22. ECL intensity of the BTD-TPA film in air during the pumping of the solution for 360 s with the streaming potential device using tap water as the solvent containing only 1 mM TPrA (dark blue line) and 1 mM TPrA with the addition of 0.5 M NaCl (light orange line) to simulate water with high salinity. Flow rate was 1.0 mL min^{-1}. Phenolic resin monolith no. 2 was employed as the filling material. PMT bias = 1000 V.

21. Supplementary references

(1) Hasegawa, G., Deguchi, T., Kanamori, K., Kobayashi, Y., Kageyama, H., Abe, T. & Nakanishi, K. High-level doping of nitrogen, phosphorus, and sulfur into activated carbon monoliths and their electrochemical capacitances. *Chem. Mater.* **27**, 4703−4712 (2015).

(2) Li, Z., Qin, W., Wu, J., Yang, Z., Chi, Z. & Liang, G. Bright electrochemiluminescent films of efficient aggregation-induced emission luminogens for sensitive detection of dopamine. *Mater. Chem. Front.* **3**, 2051−2057 (2019).

(3) Li, Z., Qin, W. & Liang, G. A mass-amplifying electrochemiluminescence film (MAEF) for the visual detection of dopamine in aqueous media. *Nanoscale* **12**, 8828−8835 (2020).

(4) Kanoufi, F., Zu, Y. & Bard, A. J. Homogeneous oxidation of trialkylamines by metal complexes and its impact on electrogenerated chemiluminescence in the trialkylamine/$Ru(bpy)_3^{2+}$ system. *J. Phys. Chem. B* **105**, 210−216 (2001).

(5) Liu, G., Nguyen, W.(H. C. H.) & Henni, A. Experimental determination of dissociation constants (pK_a) for N-(2-aminoethyl)-1,3-propanediamine, 2-methylpentamethylene diamine, N,N-dimethyldipropylenetriamine, 3,3'-diamino-N-methyldipropyl-amine, Bis[2-(N,N-dimethylamino)ethyl]ether, 2-[2-(dimethyl-amino) ethoxy] ethanol, 2-(dibutylamino) ethanol, and N-propylethanol-amine and modeling with artificial neural network. *AIChE J.* **69**, e17923 (2023).

(6) Yin, X.-B., Sha, B.-B., Zhang, X.-H., He, X.-W. & Xie, H. The factors affecting the electrochemiluminescence of Tris(2,2'-bipyridyl)ruthenium(II)/tertiary amines. *Electroanalysis* **20**, 1085−1091 (2008).

(7) Yokohama city Kawai purification plant.
