# Supplementary material for: An electrochemiluminescence device powered by streaming potential for the detection of amines in flowing solution
Source: Nat Commun. 2025 Sep 8;16:8217. doi: 10.1038/s41467-025-63548-2 (PMC12417550; doi:10.1038/s41467-025-63548-2)
Supplement: Supplementary file 2 — Description of Additional Supplementary Files [file 41467_2025_63548_MOESM2_ESM.pdf]

Description of Additional Supplementary Files

Supplementary Movie 1. ECL emission using the streaming potential device by flowing an electrolyte-free MeCN/H_2O (3:1 v/v) solution containing 1 mM TPrA at the flow rate of 0.30 mL min^{-1} and by using the phenolic resin monolith no. 1 as the filling material.
